# Postnatal Gut Immunity and Microbiota Development Is Minimally Affected by Prenatal Inflammation in Preterm Pigs

**DOI:** 10.3389/fimmu.2020.00420

**Published:** 2020-03-19

**Authors:** Xiaoyu Pan, Du Zhang, Duc Ninh Nguyen, Wei Wei, Xinxin Yu, Fei Gao, Per T. Sangild

**Affiliations:** ^1^Comparative Pediatrics and Nutrition, Department of Veterinary and Animal Sciences, Faculty of Health and Medical Sciences, University of Copenhagen, Copenhagen, Denmark; ^2^Lingnan Guangdong Laboratory of Modern Agriculture, Genome Analysis Laboratory of the Ministry of Agriculture, Agricultural Genomics Institute at Shenzhen, Chinese Academy of Agricultural Sciences, Shenzhen, China; ^3^Department of Neonatology, Rigshospitalet, Copenhagen, Denmark; ^4^Department of Pediatrics, Odense University Hospital, Odense, Denmark

**Keywords:** chorioamnionitis, small intestine, gene expression, immunity, gut microbiota

## Abstract

Chorioamnionitis (CA), resulting from intra-amniotic inflammation, is a frequent cause of preterm birth and exposes the immature intestine to bacterial toxins and/or inflammatory mediators before birth via fetal swallowing. This may affect intestinal immune development, interacting with the effects of enteral feeding and gut microbiota colonization just after birth. Using preterm pigs as model for preterm infants, we hypothesized that prenatal exposure to gram-negative endotoxin influences postnatal bacterial colonization and gut immune development. Pig fetuses were given intra-amniotic lipopolysaccharide (LPS) 3 days before preterm delivery by cesarean section and were compared with littermate controls (CON) at birth and after 5 days of formula feeding and spontaneous bacterial colonization. Amniotic fluid was collected for analysis of leukocyte counts and cytokines, and the distal small intestine was analyzed for endotoxin level, morphology, and immune cell counts. Intestinal gene expression and microbiota were analyzed by transcriptomics and metagenomics, respectively. At birth, LPS-exposed pigs showed higher intestinal endotoxin, neutrophil/macrophage density, and shorter villi. About 1.0% of intestinal genes were affected at birth, and *DMBT1*, a regulator of mucosal immune defense, was identified as the hub gene in the co-expression network. Genes related to innate immune response (*TLR2, LBP, CD14, C3, SFTPD*), neutrophil chemotaxis (*C5AR1, CSF3R, CCL5*), and antigen processing (MHC II genes and *CD4*) were also affected, and expression levels correlated with intestinal neutrophil/macrophage density and amniotic fluid cytokine levels. On day 5, LPS and CON pigs showed similar sensitivity to necrotizing enterocolitis, endotoxin levels, morphology, immune cell counts, gene expressions, and microbiota composition (except for difference in some low-abundant species). Our results show that CA markedly affects intestinal genes at preterm birth, including genes related to immune cell infiltration. However, a few days later, following the physiological adaptations to preterm birth, CA had limited effects on intestinal structure, function, gene expression, bacterial colonization, and necrotizing enterocolitis sensitivity. We conclude that short-term, prenatal intra-amniotic inflammation is unlikely to exert marked effects on intestinal immune development in preterm neonates beyond the immediate neonatal period.

## Introduction

When the newborn intestine is exposed to milk and large amount of microbes just after birth, it must be able to mount an effective immune response and tolerance against pathogens and food antigens. In the adult intestine, this homeostasis is maintained by a structured and rapidly renewing epithelium that is reinforced by various aspects of the innate and adaptive immunity (1). For neonates, and especially preterm neonates, the situation is different. Before birth, the intestine is bathed in sterile amniotic fluid through fetal swallowing, contributing up to 20% of fetal energy and protein supply (2). Upon delivery, the neonatal intestine encounters a new microbe-rich environment with oral intake of milk. Meanwhile, both innate and adaptive immune systems still undergo differentiation and adaptation (3). Immaturity-related disruption of the equilibrium may lead to disease susceptibility. Thus, necrotizing enterocolitis (NEC) occurs in many preterm infants during the first weeks of life, and NEC is associated with microbiota dysbiosis and inappropriate immune response (4) with short- and long-term consequences (5, 6). It is important to better understand how the newborn preterm gut develops its immune competence, interacting with the gut microbiota and prenatal insults, to better prevent harmful inflammatory reactions in the postnatal period.

Chorioamnionitis (CA), caused by intra-amniotic inflammation, is a common cause of preterm birth (7). The CA-related inflammatory *in utero* environment may pose an additional challenge for the fetal immature intestine via swallowing of amniotic fluid (8). Using fetal lambs and sheep and rodents, it has been shown that intra-amniotic inflammation affects the neonatal intestine with regard to immune cell infiltration, tight junction proteins, and villus structure (9–13). These studies mainly investigated intestinal outcomes around birth, without demonstrating if such effects were persistent, or even worsened, when transitioning into postnatal life when the normal developmental changes occur associated with hemodynamic stability, enteral nutrition, and gut bacterial colonization within the first 1–2 weeks. It is unknown if these fundamental postnatal adaptations overshadow any effect of intestinal exposure to bacteria, bacterial toxins, and inflammation *in utero*. Prenatal gut inflammation may also influence the postnatal colonization with bacteria and thereby influence intestinal immune development.

Over the past decades, the preterm pig has been extensively used as a valuable biomedical model for preterm infants because of its similarity in size, anatomy, and birth-related clinical complications, such as impaired lung, immunity, gut, and brain functions (14). When kept in a neonatal intensive care unit under high-sanitary conditions, thermoregulation, and nutrition and oxygen support, preterm pigs develop to levels of the their term counterparts within 3–4 weeks, although some functions lack behind for much longer (15). The pig model is generally used to investigate preterm birth independent of the factors leading to preterm birth (e.g., maternal inflammation), but we have recently investigated the specific role of CA, induced by intra-amniotic lipopolysaccharide (LPS) exposure, on organ development in preterm neonates (16). Further building on this model, we hypothesized in this study that exposure of the fetus and the fetal gut (via swallowing of amniotic fluid) to some days of LPS before preterm delivery would influence intestinal gene expressions, not only at birth, but also beyond the neonatal period, after reaching physiological stability and adapting to gut bacterial colonization. By intestinal transcriptome and metagenome profiling, our data showed that CA-like symptoms induced by fetal LPS exposure increased the expression of a number of intestinal genes associated with immune cell infiltration at birth but that these fetal CA symptoms were marginal a few days later, following the start of enteral feeding and spontaneous gut bacterial colonization.

## Materials and Methods

### Animal Procedure and Tissue Analysis

All animal procedures were approved by the Danish National Committee on Animal Experimentation. Details of animal procedures were described previously (16). Briefly, fetuses from 3 sows were assigned to LPS treatment group (LPS, *n* = 37) or control group (CON, *n* = 32), respectively. In the LPS group, each fetus received 1 mg LPS (*Escherichia coli* 055:B5; Sigma-Aldrich, St. Louis, MO, USA) into the amniotic fluid, and fetuses in the CON group received saline or no injection. Preterm piglets were born at day 106 of gestation (3 days after injection) by cesarean section. During the cesarean section, amniotic fluid was collected for analysis of total leukocyte counts (by manual counting under a microscope) and cytokines (by enzyme-linked immunosorbent assay). For each group, piglets were randomly assigned to be euthanized within 1–3 h of birth or to receive parenteral nutrition plus supplemental enteral nutrition with infant formula for 5 days. The formula composition included 75 g/L Liquigen MCT (Nutricia, Allerød, Denmark), 80 g/L Pepdite (Nutricia), and 70 g Lacprodan DI-9224 (Arla Foods Ingredients, Viby, Denmark). The piglets received a gradually increasing amount of infant formula (3–15 mL/kg per 3 h) for 5 days, a feeding protocol leading to ~50% of pigs with macroscopically visible NEC lesions in the colon region (16).

From the above fetuses, animals that died *in utero* or within the first 48 h because of respiratory distress were excluded in the study. A subsample of 40 individuals were randomly chosen from the remaining animals for the phenotypic, transcriptomic, and metagenomic analyses in this study and balanced among litters and sexes. Four groups of pigs were sampled either within 1–3 h of delivery (day 1) or on postnatal day 5 and subjected to LPS or control treatment before birth (CON-D1, LPS-D1, CON-D5, LPS-D5, each *n* = 10). The distal small intestines were collected and analyzed for endotoxin levels, morphology (i.e., villus height and crypt depth), brush border enzyme activities, and goblet cell and immune cell counts (including CD3-, Foxp3-, and MPO-positive cells) by immunohistochemistry as previously described (16). Comparisons were made to examine the prenatal LPS effects on the distal intestine at birth (samples collected on day 1 within 1–3 h after delivery) and on day 5, respectively (i.e., LPS-D1 vs. CON-D1 and LPS-D5 vs. CON-D5). Because the phenotypic difference between D1 and D5 pigs involves many more factors than postnatal age alone (e.g., feeding responses, metabolic adaptation, bacterial colonization), no attempts were made to investigate specific age × treatment interactions.

### RNA-Seq Analysis

Total RNA from the distal small intestines was isolated with RNeasy Micro Kit (Qiagen), and 1.5 μg RNA per sample was used for RNA-seq library construction. Sequencing libraries were constructed using NEBNext Ultra RNA library Prep Kit for Illumina (NEB, Ipswich, MA, USA) following the manufacturer's recommendations. RNA libraries were sequenced on the Illumina HiSeq 4000 platform with paired-end 150-bp reads production. Quality and adapter trimming of raw reads was performed using TrimGalore (Babraham Bioinformatics, Cambridge, UK). The remaining clean reads were aligned to the porcine genome (Sscrofa11.1) using Tophat (17). The annotated gene information of porcine genome was downloaded from Ensembl. The script *htseq-count* (18) was used to generate gene count matrix, followed by gene-level differential expression analyses using *DESeq2* (19).

### Metagenome Analysis

Total genomic DNA from distal intestinal contents was extracted using CTAB/SDS method. Sequencing libraries were generated using TruSeq DNA PCR-Free Sample Preparation Kit (Illumina, USA) following manufacturer's recommendations. The library was sequenced on an Illumina HiSeq Xten platform, and 150-bp paired-end reads were generated. Clean reads after removing host sequence were employed to contigs assembly using MEGAHIT (v1.0.6) (20). Open Reading Frames (ORFs) were identified by MetaGeneMark (v3.38) (21), and the ORFs were clustered for removing redundancy by CD-HIT (v4.7) (22). Taxonomic information of ORFs was annotated by DIAMOND (23) based on pig microbial database (24). Functional information of ORFs was annotated by KAAS (KEGG Automatic Annotation Server) (25) against KEGG database and by DIAMOND against COG (Clusters of Orthologous Groups) and CAZy (carbohydrate-active enzymes) database.

### Immunohistochemistry Analysis

Paraformaldehyde- or acetone-fixed distal small intestine sections were examined by immunohistochemistry for DMBT1 (anti-DMBT1, HPA040778; Sigma), SFTPD [mouse anti–pig surfactant protein D (SP-D), MCA2725; Bio-Rad, Hercules, CA, USA] and MHCII (mouse anti–pig SLA CLASS II DQ, MCA1335GA; Bio-Rad). Staining was developed with UltraVision LP Detection System (ThermoFisher Scientific, Waltham, MA, USA). The sections were counterstained with Mayer hematoxylin. Images were acquired using the Leica LAS EZ software (version 3.4.0), Wetzlar, Germany, and the proportion of positive staining was analyzed by the IHC toolbox in ImageJ, National Institutes of Health, USA.

### Statistical Analysis

Using R software package (version 3.5.1), Vienna, Austria, amniotic fluid and tissue measures were analyzed by using linear model (lm function), adjusted for litter. The linearity of data, normality of residuals, and homoscedasticity were checked, and data were transformed using Box-Cox transformation when required. *P* < 0.05 was considered statistically significant; *P* < 0.10 was considered a tendency to an effect. For RNA-seq analysis, significant differentially expressed genes (DEGs) with fold change >2 and Benjamini-Hochberg (BH)–adjusted *P* < 0.05 were identified by DESeq2 and adjusted for litter. Correlation between gene expression was performed using Spearman rank correlation based on normalized counts produced from DESeq2, and the correlation with absolute Spearman ρ >0.6 as well as BH-adjusted *P* < 0.05 was considered statistically significant. Gene Ontology and KEGG pathway enrichment analysis were performed using DAVID (26), and BH-adjusted *P* < 0.05 was considered statistically significant. Validation of DEGs was performed by immunohistochemistry analysis, where Wilcoxon rank-sum test was used for the difference test, and *P* < 0.05 was considered statistically significant. For metagenome, diversity analysis was performed based on normalized taxonomy abundance. The difference test of alpha diversity was performed using Wilcoxon rank-sum test, and *P* < 0.05 was considered statistically significant. Analysis of similarities was used to check the difference in beta diversity (based on Bray–Curtis dissimilarity matrix), and *P* < 0.05 was considered statistically significant. Differentially abundant features at different levels were analyzed by Metastats (27), and *P* < 0.05 was considered statistically significant. Differential abundant genes, KEGG pathway, and COGs and CAZymes were identified by the Wilcoxon rank-sum test with BH-adjusted *P* < 0.05.

## Results

### Prenatal LPS Exposure Induces Intra-Amniotic Inflammation and Intestinal Immune Cell Infiltration at Birth

Intra-amniotic LPS injection induced extensive intra-amniotic inflammation, with increased leukocytes and cytokines [interleukin 1b (IL-1b), IL-6, IL-8, IL-10, and tumor necrosis factor α (TNF-α)] in the amniotic fluid (all *P* < 0.05, Table S1). Focusing on the distal small intestines of these neonates (Table S2), we found that shortly after birth (day 1) the LPS-exposed preterm pigs had elevated gut endotoxin level (*P* < 0.01) and tended to have reduced villus height (*P* < 0.1), whereas crypt depth was not affected. There were limited effects of LPS on digestive functions, as measured by lactase, maltase, and sucrase activities. To investigate immune cell infiltration in response to LPS exposure, the distal small intestines were stained for CD3, Foxp3, and myeloperoxidase (MPO, marker of neutrophil/macrophage infiltration). The results showed that the LPS-exposed preterm pigs had increased MPO-positive cell density in the distal small intestines on day 1 (*P* < 0.05). Goblet cells were quantified by Alcian blue and periodic acid–Schiff staining and showed no difference between the LPS and CON groups on day 1. After 5 days, pigs with and without prenatal LPS exposure had similar incidence of intestinal NEC lesions, and all the above measures in the distal small intestine were no longer different between the LPS and CON groups (Table S2).

### Prenatal LPS Exposure Up-Regulates Intestinal Gene Expression at Birth

**Figure 1:**
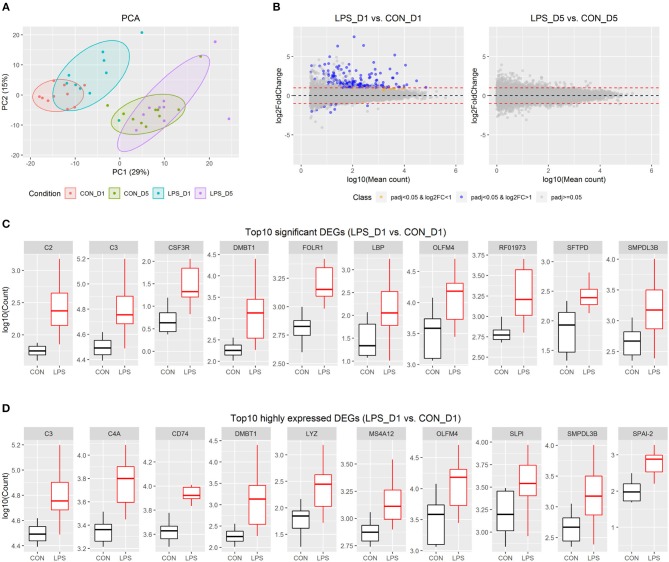
Intestinal transcriptome profile in response to intra-amniotic LPS exposure. **(A)** Scores of the first two principle components from principal component analysis are plotted, with ellipses for the 80% confidence interval for each group. **(B)** MA plot showing fold change (LPS vs. CON) and mean of normalized counts for all the analyzed genes. **(C)** Boxplot of top 10 significant DEGs between LPS-D1 and CON-D1 groups. **(D)** Boxplot of top 10 highly expressed DEGs between LPS-D1 and CON-D1 groups.

The distal small intestinal tissues were used for RNA-seq, and approximately 48 M reads were sequenced for each pig. Of 25,880 pig genes annotated in the Ensembl database (Sscrofa11.1), 20,874 genes were detected in at least one pig and were used for further analysis. Based on the individual expression level of these genes, principal component analysis showed that intestinal gene expression shortly after birth (i.e., day 1) was distinct from that in the postnatal period (i.e., day 5). In addition, there were genes showing moderate variation between the LPS and CON groups on day 1, but not on day 5 (Figure 1A).

To detect the changes in gene expression between the LPS and CON groups on days 1 and 5, respectively, we employed a threshold with fold-change >2 and adjusted *P* < 0.05 to identify significant DEGs using DESeq2. The results showed that 176 of 20,874 genes (equivalent to 0.8% of genes analyzed) were identified as DEGs between the two groups on day 1, whereas no significant DEGs were found on day 5 (Figure 1B and Table S3). The DEGs on day 1 included 166 up-regulated and 10 down-regulated genes, respectively. Based on the current gene annotation in the pig genome, 129 genes with gene symbols were ascertainable according to the Ensembl database. Except for *ADGRG2, NEK5, ssc-mir-421*, and 7 other genes without gene symbols, all other DEGs showed up-regulation in the LPS vs. CON group on day 1. The top 10 most significant genes were *DMBT1, LBP, CSF3R, SMPDL3B, FOLR1, SFTPD, C3, C2, RF01973*, and *OLFM4* (Figure 1C). *C3, DMBT1, OLFM4*, and *SMPDL3B* were also among the top 10 DEGs with highest average expression across all pigs on day 1 (Figure 1D).

### Prenatal LPS Exposure Has Limited Effects on Gut Microbiome Postnatally

**Figure 2:**
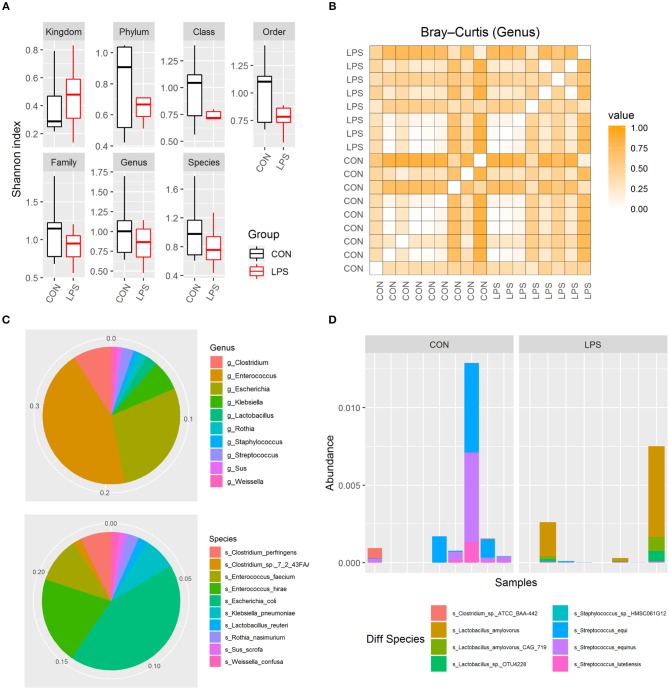
Gut microbiome in LPS and CON groups on postnatal day 5. **(A)** Boxplot of Shannon index in two groups. **(B)** Heatmap derived from dissimilarity matrix of Bray–Curtis distances from all 5-day-old pigs. **(C)** Pie chart of top 10 abundant genus/species in all 5-day-old pigs. **(D)** Bar plot showing eight differential abundant species between the LPS and CON groups.

To investigate whether intra-amniotic LPS exposure affected the neonatal gut colonization, 5-day-old formula-fed preterm pigs had their distal small intestinal content collected for metagenome analysis. Based on Shannon index and Bray–Curtis dissimilarity, the alpha and beta diversities of gut microbiome were not affected by intra-amniotic LPS exposure, regardless of taxonomic levels (Figures 2A,B; all *P* > 0.05). Similar to that in preterm human infants in the first few days after birth (28, 29), these 5-day-old preterm pigs were dominated by phylum Proteobacteria and Firmicutes (mean abundance was 44.2 and 26.6%, respectively). The top abundant genus included *Enterococcus, Escherichia*, and *Clostridium* (mean, 17.2, 11.0, and 3.5%, respectively), and the top abundant species were *E. coli, Enterococcus hirae*, and *Enterococcus faecium* (mean, 10.6, 5.0, and 2.6%, respectively; Figure 2C). The abundance of these features showed no statistical difference between the two groups. However, eight differentially abundant species were identified between the LPS and CON groups (*P* < 0.05) according to Metastats and were detected in at least three pigs (Figure 2D). The mean abundance of these eight species was relatively low, and *Streptococcus equi* and *Lactobacillus amylovorus* were among the most abundant (~0.05%). Their maximum individual abundance was ~0.58%. These two species were down- and up-regulated in the LPS group, respectively. Moreover, 897 of 21,384 analyzed genes showed differential abundance between the two groups (adjusted *P* < 0.05), and most of them (891/897) were down-regulated in the LPS group. Approximately 11% of the down-regulated genes were annotated to *Streptococcus*, and their top COG annotations were “Replication, recombination, and repair,” “Carbohydrate transport and metabolism,” and “Defense mechanisms.” However, the abundance of these functions was not significantly different between the two groups.

### Prenatal LPS Exposure Induced Intestinal Immune Response Associated With DMBT1

**Figure 3:**
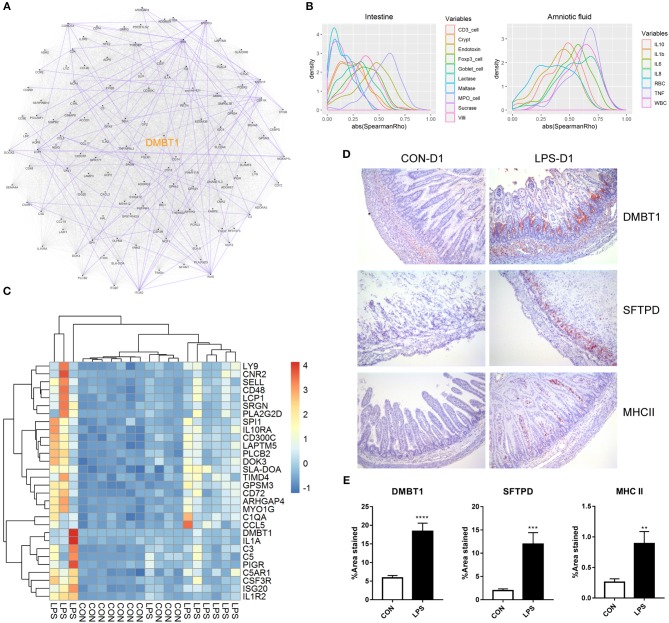
Relationship and functionality of DEGs between LPS and CON groups at birth. **(A)** Putative co-expression network, where each node represents a gene, and each edge represents a significant correlation between gene pairs. Experimentally determined protein–protein interaction between gene pairs is highlighted in purple. Hub gene DMBT1 is highlighted in yellow. **(B)** Density plot of absolute Spearman ρ between DEGs and intestinal/amniotic measures. **(C)** Heatmap showing the relative expression of DEGs that were correlated with intestinal MPO-positive cell density and amniotic TNF-α level. **(D)** Representative images of distal small intestines stained with antibodies to DMBT1, SFTPD, and MHCII. **(E)** Significant difference of the area stained by DMBT1/SFTPD/MHCII between LPS and CON groups was revealed according to Mann–Whitney *U* test. Values are presented as the mean ± SEM, *P < 0.05, **P < 0.01, ***P < 0.001, ****P < 0.0001.

Finally, to better understand the relationship of the DEGs identified on day 1, we first conducted a putative co-expression gene network using pairwise correlation to determine possible gene pairs in the expression data. Based on the 129 DEGs with ascertainable gene symbols, 4,205 of 8,256 gene pairs were selected using a threshold of absolute Spearman ρ > 0.6 and adjusted *P* < 0.05, and were used to build the co-expression network. Next, to identify the highly connected gene (hub gene) in this network, network analysis was performed using Cytoscape. *DMBT1* (deleted in malignant brain tumors 1) was identified as the hub gene as it had the highest “betweenness centrality,” which describes the shortest-path connectors through a network (Figure 3A). In addition, experimentally determined protein–protein interaction between these gene pairs was examined using STRING database. We found that DMBT1 interacts with SFTPD (SP-D) and that SFTPD interacts with TLR2 (Toll-like receptor 2), all of which were up-regulated in the newborn pigs exposed to intra-amniotic LPS.

To investigate the biological processes and pathways associated with the DEGs, we performed functional enrichment analyses using the web tool “DAVID” and found that the DEGs identified on day 1 were related to “innate immune response,” “neutrophil chemotaxis,” “complement and coagulation cascades,” “cell adhesion molecules (CAMs),” and “antigen processing and presentation” (Table S4). Thus, both innate and adaptive immune responses were involved in the neonatal intestine exposed to intra-amniotic LPS. Differentially expressed genes involved in the innate immunity were related to pattern recognition (*TLR2, CD14, LBP, DMBT1, SFTPD*), complement system (*C1QA, C1QB, C1QC, C1RL, C2, C3, C4A, C5*) and neutrophil chemotaxis (*C5AR1, CSF3R, CCL5, CCL17, CCL19*). For the adaptive immunity, five DEGs were enriched in the KEGG pathway “antigen processing and presentation,” encoding for major histocompatibility complex (MHC) classes I and II, as well as CD4. Consistent with that the recruitment of CD4^+^ T cells in the mice neonatal small intestine is based on β7 integrin–dependent pathways (30), *ITGB7* (integrin beta-7) was also found to be one of the DEGs and was up-regulated in the LPS group. However, the mRNA expression of co-stimulatory molecules for T-cell activation (i.e., CD80, CD86, CD28, and ICOS), as well as T-cell transcription factors (i.e., T-bet for T_H_1, GATA3 for T_H_2, RORγt for T_H_17, and Foxp3 for Tregs cells), showed no significant difference between the LPS and CON groups.

In addition, pairwise correlation test between each DEG and the measures in the amniotic fluid or small intestine was performed. Consistent with that the DEGs identified on day 1 were enriched in innate immune response and neutrophil chemotaxis as mentioned previously, the correlation test showed that most of the DEGs correlated with the MPO-positive cell density in the small intestine (mean absolute Spearman ρ = 0.54; Figure 3B). The DEGs were also correlated with cytokine levels, especially TNF-α, in the amniotic fluid (mean absolute Spearman ρ = 0.62; Figure 3B). After multiple testing, 44 and 76 DEGs were significantly and positively correlated with the MPO-positive cell density in the small intestine and TNF-α level in amniotic fluid (adjusted *P* < 0.05, mean ρ = 0.69 and 0.71, respectively). Genes significantly correlated with both intestinal MPO-positive cell density and amniotic TNF-α (Figure 3C) included *DMBT1*, the hub gene in the putative co-expression network, and those involved in complement system (*C1QA, C3, C5*) and neutrophil chemotaxis (*C5AR1, CSF3R, CCL5*), suggesting an underlying mechanism whereby intra-amniotic inflammation affects intestinal immune response via fetal swallowing.

To examine whether the identified DEGs were altered also at the protein level, immunohistochemistry analysis was performed for selected proteins in the newborn pigs (D1 samples). This included DMBT1, the hub gene in the co-expression network, and SFTPD that might interact with DMBT1, both of which are related to innate immunity. In addition, MHCII, related to adaptive immunity, was examined. The results showed that DMBT1 and SFTPD were mainly expressed in the crypts of the distal small intestine and up-regulated in the LPS vs. CON group (mean positive area = 18.4 vs. 5.9% for DMBT1, and 12.0 vs. 2.0% for SFTPD, both *P* < 0.05). MHCII, on the other hand, was distributed within the villi of distal small intestine and also showed higher expression in the LPS vs. CON groups (mean positive area = 0.9 vs. 0.3%, *P* < 0.05; Figures 3D,E).

## Discussion

Perinatal development of the small intestine, including its immune system, occurs as the combined result of an intrinsic (genetic, endocrinological) program and environmental factors including oral nutrition/fluid (e.g., amniotic fluid before birth, milk after birth) and exposure to microbiota (e.g., infected amniotic fluid before birth, bacterial colonization after birth). The maturational pattern is partly species- and birth-dependent, and we have previously shown that in pigs the small intestine has a remarkable capacity to mature over the first weeks following the initial deficits in response to preterm birth. We now show that prenatal exposure to inflammation *in utero* induces altered expression of both innate and adaptive immunity-associated genes in the neonatal small intestine, together with marked immune cell infiltration at birth. However, after introduction of enteral feeding and ongoing bacterial colonization, intestinal gene expression, and functions (NEC susceptibility, morphology, digestive enzymes, global gene expression) were similar on day 5 in control and LPS-exposed preterm pigs. In addition, prenatal inflammation had a minor effect on the gut colonization at this time after birth (e.g., the low-abundant *S. equi* and *L. amylovorus* species were reduced and increased, respectively, in LPS-exposed pigs on day 5). Collectively, our results confirm that the birth- and age-related development of intestinal microbiota and gene expressions are remarkably resilient to exposure of a few days of intra-amniotic inflammation before preterm birth. In a translational perspective, intestinal functions of preterm infants born after acute CA may therefore not be markedly compromised in the postnatal period, relative to infants born prematurely without prenatal inflammation. Future studies should address if this conclusion is true also for other periods, doses and types of prenatal inflammatory insults (e.g., using common CA-related pathogens such as *Ureaplasma*), as well as longer postnatal age and interacting factors (e.g., feeding modes, antibiotics, infections).

In the present study, we identified a number of genes that might be co-expressed and up-regulated in the neonatal small intestine exposed to fetal inflammation. The hub gene of this putative co-expression network was *DMBT1*, which is a pattern recognition receptor localized to epithelial cells and binds to a broad range of pathogens (31, 32). Our results show that the intestinal *DMBT1* was up-regulated by prenatal LPS exposure, both at mRNA and protein levels. *DMBT1* in the intestinal epithelial cells (IECs) plays an important role in first-line defense by preventing bacterial invasion into the IECs and inhibiting cytokine secretion (33). Loss of *DMBT1* in mice leads to enhanced dextran sulfate sodium–induced colitis (34). Thus, up-regulation of *DMBT1* in the neonatal intestine in response to CA might play a role in intestinal mucosal protection. In addition to immune cell infiltration, villus structure was moderately compromised around birth following prenatal LPS exposure. Like all the other measures in the small intestine, such effects induced by prenatal LPS exposure disappeared after 5 days. This could be related to *DMBT1*, which negatively affects epithelial cell growth (35) and is associated with a change from proliferation to differentiation in the epithelium and thereby epithelial regeneration following mucosal damage (36).

DMBT1 also binds to mucosal defense proteins, such as SP-D (37). Surfactant protein D exerts antimicrobial effects by bacterial agglutination, enhancing their clearance by phagocytic cells (38). Similar to DMBT1, lack of SP-D in mice also showed increased susceptibility to dextran sulfate sodium–induced colitis (39). Moreover, both DMBT1 and SP-D have been shown to stimulate alveolar macrophage migration (40, 41), suggesting a role of DMBT1 and SP-D in mediating the cross-talk between epithelial cells and the underlying immune cells. Such cross-talk may also exist in the gastrointestinal tract, and MPO-positive cells were increased in density in parallel with the up-regulation of DMBT1 and SP-D, following intra-amniotic endotoxin exposure. Functional enrichment analysis using all the DEGs also revealed pathways related to CAMs and chemokine signaling pathway, which might be relevant to the macrophage migration.

Apart from innate immunity, adaptive immunity may be also involved in the neonatal small intestine in response to CA. We found that genes in the “antigen processing and presentation” pathway were up-regulated, including those that encode for MHC class II and CD4. The MHC molecules are normally expressed on antigen-presenting cells (APCs) such as macrophages, but they are also found in the upper villus under normal physiologic conditions and in the crypts in case of disease (42). By immunohistochemistry analysis, we found that intra-amniotic endotoxin up-regulated MHCII expression that was mainly distributed inside the villi, which suggests CA effects on classic APCs. Our transcriptome data showed that *CD4* was also up-regulated in the LPS-exposed neonatal intestine. However, given the absence of up-regulation of co-stimulatory molecules and T-cell transcription factors in our transcriptome data, it remains unclear if CA activates CD4 T cell in the neonatal intestine. In the lung, T-cell activation could be inhibited by SP-D (43). It remains to be investigated whether this mechanism also exists in the gastrointestinal tract. Nevertheless, in both human and mice intestines, CD4^+^ T cells are generally naive in the neonatal period (30, 44), and their response to prenatal endotoxin exposure requires further investigation.

There are limitations related to our experimental conditions used to reflect human CA. Thus, our inflammatory insults were induced by a few days of intra-amniotic LPS rather than by giving one or more CA-associated pathogens at various times and doses. Hence, our experimental conditions may not accurately reflect all human CA conditions. It has been shown that the organisms commonly identified in CA include *Ureaplasma urealyticum*, group B *Streptococcus*, and *E. coli*, but it remains unclear whether these are causative agents or only bystanders to the inflammatory effects (45). Thus, in this study, we decided to use intra-amniotic LPS to induce a highly controlled, relatively short-term inflammation of the fetal membranes and evaluated its effects on the neonatal gut independent of source of pathogens. Our result showed increased intestinal MPO, *CD4*, and *TLRs* in response to such prenatal inflammation, which are similar to those that resulted from *Ureaplasma* infection in sheep model (10), suggesting common underlying mechanisms. On the other hand, such intra-amniotic LPS model limits our analysis and interpretation of gut microbiome. During healthy pregnancy, the fetal environment is sterile, and neonatal gut colonization starts in the hours postpartum, whereas for CA neonates, the gut microbial colonization may have started already before birth through ingestion of infected amniotic fluid (46). Thus, CA neonates may harbor a different initial gut microbiome than healthy infants. We studied here the responses to prenatal inflammation, independently of such pathogen exposure and hence assessed only the postnatal environmentally derived colonization. We show that this colonization is not notably affected by prenatal inflammation. It remains to be investigated how and for how long the gut microbiota may be affected in human preterm infants exposed to pathogens before birth via swallowing amniotic fluid. Another limitation is that in our control group not all the fetuses received saline injection to avoid unnecessary prolonged surgery. However, the pigs with and without saline injection were combined into a joint control group as there was no difference in any amniotic fluid or intestinal phenotypes (parameters listed in Tables S1, S2) between them. We cannot completely exclude a small effect of the intra-amniotic saline injection, although such minimal manipulation of the uterus and fetus is unlikely to affect the overall pronounced effects of LPS on gut gene expression on D1.

In summary, we characterized the intestinal gene expression and microbiome in a preterm pig model of CA. We found that intra-amniotic LPS exposure affected the expression of intestinal genes in preterm pigs at birth, especially genes related to immune cell infiltration. Following enteral feeding and bacterial colonization, intra-amniotic LPS had limited effects on intestinal structure and function. A short period of intra-amniotic inflammation prior to preterm birth is unlikely to cause longer-lasting pro-inflammatory responses in the gut of preterm infants. This does not exclude, however, that CA with a longer duration of inflammation before birth, and with more aggressive types and doses of pathogens, could have long-term detrimental effects on the developing intestine in preterm infants.

## Data Availability Statement

The datasets generated for this study can be found in the Gene Expression Omnibus, accession number GSE139366, and EBI Metagenomics, accession number PRJEB34982.

## Ethics Statement

The animal study was reviewed and approved by Danish National Committee of Animal Experimentation.

## Author Contributions

XP and DZ analyzed and interpreted the transcriptome and metagenome data. XP was the major contributor in writing the manuscript. WW and XY performed the transcriptome library construction. PS, FG, and DN took part in the main study design and critically reviewed the manuscript. All authors read and approved the final manuscript.

### Conflict of Interest

The authors declare that the research was conducted in the absence of any commercial or financial relationships that could be construed as a potential conflict of interest.
